# Aberrant Auditory Steady-State Response of Awake Mice Induced by Chronic Interferon-α Treatment

**DOI:** 10.3389/fphar.2020.584425

**Published:** 2021-01-27

**Authors:** Yingzhuo Li, Xuejiao Wang, Jingyu Chen, Zijie Li, Pingting Yang, Ling Qin

**Affiliations:** ^1^ Department of Physiology, China Medical Univeristy, Shenyang, China; ^2^ Department of Rheumatology and Immunology, First Affiliated Hospital, China Medical University, Shenyang, China

**Keywords:** interferon-alpha, depressive-like behavior, microglia, astrocytes, auditory cortex, EEG

## Abstract

**Background:** Patients receiving the cytokine immunotherapy of interferon-alpha (IFN-α) frequently present with depression. This is one of the excellent models to explore the action of peripheral cytokine on central nervous system (CNS) and to study the development of depression. The auditory steady-state response (ASSR), electroencephalogram (EEG) oscillations induced by periodic acoustic stimulation, is an effective approach to evaluate the neural function in mental illness including depression. The aim of the present study was to investigate the effect of IFN-α on the cortical ASSR and its correlation with depressive-like behavior.

**Methods:** Chronic electrodes were implanted on the skull over the auditory cortex (AC) of male C57BL/6 mice. The animals were treated with daily injection of IFN-α or saline (vehicle) for three weeks. EEGs were recorded in AC of the same mouse before and after the injection treatment to monitor the changes of ASSR induced by IFN-α. Depressive-like behavior was analyzed in the forced swim test (FST). Immunohistochemical staining was used to examine the status of neuron and glia in the hippocampus and AC.

**Results:** Compared to pretreatment condition, injection of IFN-α significantly reduced the power of 40 Hz ASSR in the mouse AC from the second week. Such a decrease continued to the third week. The immobility times of FST were significantly increased by a 3-week treatment of IFN-α and the immobility time was negatively correlated with the power of 40 Hz ASSR. Astrocytes and microglia in the hippocampus and AC were activated by IFN-α, but the density of neuron was not significantly affected.

**Conclusion:** Our results suggest that EEG measurement of ASSR may be used as a biomarker to monitor the CNS side effects of IFN-α treatment and to search a novel intervention with potential therapeutic implications.

## Highlights

IFN-α reduces the power of ASSR in the cortex of mice. IFN-α induces depressive-like behavior in mice. Reduction of ASSR is correlated with depressive-like behavior. ASSR deficit is accompanied with glia activation in the hippocampus and cortex.

## Introduction

Interferon-alpha (IFN-α) is an innate immune cytokine that has both antiviral and antiproliferative activities and widely used in the immunomodulatory treatment in patients with chronic hepatitis C virus (HCV) infection (Alavi et al., 2012) or malignant melanoma (Tarhini et al., 2012). However, IFN-α treatment can cause serious side effects on the central nervous system (CNS), leading to treatment interruption (Quesada et al., 1986; Vial and Descotes, 1994). For example, the standard treatment of IFN-α with HCV infection is associated with the development of major depressive episode (MDE) in up to 45% of HCV patients (Musselman et al., 2001; Raison et al., 2005; Su et al., 2010). Depression is a serious CNS side effect which sometimes leads patients to committing suicide during IFN-α therapy (Laguno et al., 2004). Animal studies in rodents or rhesus monkeys also showed that acute or chronic exposures of IFN-α can induce depression-like behaviors (Makino et al., 2000; Sammut et al., 2001; Felger et al., 2007). However, the mechanisms by which IFN-α induces the CNS side effect are still under investigation.

IFN-α has been demonstrated to have regulatory effects on the neuronal excitabilities in CNS (Calvet and Gresser, 1979) and influence the function of neurotransmitter metabolism and neuroendocrine linking to depression (Menzies et al., 1996; Kumai et al., 2000; Kitagami et al., 2003; De La Garza et al., 2005; Felger et al., 2007). Also, IFN-α is a potent stimulator of proinflammatory cytokines not only at the periphery but also within the CNS (Borden et al., 2007). IFN-α is known to induce the production of interleukin-1 (IL-1) and tumor necrosis factor α (TNF-α), which can induce neuroinflammation (Kim et al., 2016; Na et al., 2014). Accumulating evidence suggests that pathophysiology of depression might be associated with activated inflammatory processes (Dantzer et al., 2008; Miller et al., 2009). Clinically depressed patients have been found to have higher levels of proinflammatory cytokines and inflammatory markers (Maes, 1999; Schiepers et al., 2005; Dowlati et al., 2010). To date, no coincident conclusion has been reached about whether the IFN-α induced depression-like behavior is due to the direct neuromodulation effect of IFN-α or secondary inflammation process.

Recording the electrical activity of neurons, such as electroencephalogram (EEG), is an effective mean to evaluate the CNS function. Previous studies on the spontaneous EEG activities have revealed several EEG abnormalities in both the patients during IFN-α therapy (Mattson et al., 1983; Rohatiner et al., 1983; Suter et al., 1984) and animals that received IFN-α treatment (Birmanns et al., 1990). Recently, accumulating evidence suggests that auditory steady-state response (ASSR), cortical oscillations in the gamma frequency range (40 Hz) induced by periodic acoustic stimulation, is an effective approach to evaluate the neural function in mental illness including bipolar disorder (Oda et al., 2012; Isomura et al., 2016; Zhou et al., 2018) and schizophrenia (Krishnan et al., 2009; Javitt and Sweet, 2015) and in neuropharmacological experiments on animal models (Leishman et al., 2015; Shahriari et al., 2016; Sivarao et al., 2016). EEG measurement of ASSR reflects the integrity of the sensory pathways and the capacity of these pathways to generate synchronous activity. ASSR deficits have been reported in the patients with psychosis (Oda et al., 2012; Isomura et al., 2016; Zhou et al., 2018). However, whether ASSR deficits are associated with IFN-α induced CNS side effects remains unknown.

Given the above, we determine the potential effect of IFN-α on the ASSR by conducting EEG recording on mice through the chronic electrodes implanted in the skull over the auditory cortex (AC). The AC plays a critical role in the cortical auditory processing (Dong et al., 2011; Dong et al., 2013). We recorded the EEGs in AC of the same mouse treated by IFN-α or saline (vehicle) to monitor the changes of ASSR induced by IFN-α. And the forced swimming task (FST) was used to access depressive-like behaviors of the mice, in which an increased duration of immobility signifies behavioral despair (Petit-Demouliere et al., 2005). Histomorphologic changes of neurons and glia were analyzed to test for possible linkages between behaviors, ASSR deficits, and neuroinflammation.

## Materials and Methods

### Mice

Experiments were performed using 8–12-week-old C57BL/6 male mice (Vital River Laboratory, Beijing, China). All animals were maintained in standard animal cages under conventional laboratory conditions (12/12 h light/dark cycle, 22°C) with ad libitum access to food and water. The animals were maintained and treated in compliance with the policies and procedures detailed in the “Guide for the Care and Use of Laboratory Animals” of the National Institutes of Health. The animal experimental protocols of the “Guide” and the treatment procedures were reviewed and approved by the Animal Care and Use Committee of China Medical University (No. KT2018060). All surgeries were performed under anesthesia, and all efforts were made to minimize animal suffering.

### Surgery of Electrode Implantation

Mice were handled according to the criteria of the ethics committee at our institution. Following a period of two weeks of handling for at least once a day for 5 min, animals were subjected to a surgery for implantation of chronic single-wire electrodes. Animals were kept under anesthesia during the whole procedure with a gaseous mixture of 2% isoflurane in air. Atropine sulfate (0.1 mg/kg) was used to reduce the viscosity of bronchial secretions. Temperature was monitored rectally and maintained at 37°C using a feedback-controlled blanket. After placing the animal in a stereotaxic frame (#68001, RWD Life Science, Shenzhen, China), the cranium was exposed. Two stainless screws were separately inserted into the left hemisphere of AC (AP = −2.3–3.5 mm, ML = +3.5–4.0 mm, and DV = −2–2.5 mm) according to the standard mouse stereotaxic atlas (Konsman, 2003). A sliver microwire (ID 30µm, OD, #785500, A-M Systems, Hofheim, United States) as an electrode was fixed on the bone by the screws in one end. The other end of the microwire was soldered to a pin connector, which was secured onto the cranium using dental acrylic resin. A stainless-steel screw electrode over the cerebellum served as ground. Four additional skull screws were implanted serving as anchors. Animals were allowed to recover for 1 week.

### Electrophysiological Recording and Sound Stimuli

After recovery from surgery, animals were familiarized with the sound-attenuated recording room. Briefly, the animals were transported in their home cage to the recording room where they were left alone for 5 min. They were then put in a mesh box (20 × 20 × 30 cm) and tethered to the recording system via a flexible cable for 15 min. This procedure was repeated for 4 days. Recording experiments were conducted on the fifth day. The sound stimulus used in our experiments was a train of click sounds to assess ASSR. The waveform of each click was a rectangular pulse of a 0.2 m s duration, which was repeated at a rate of 40 cycles/s and continued for 0.5 s duration. The waveforms were generated digitally with a 100 kHz sampling rate using a custom-built MATLAB (Mathworks, Natick, MA, United States) program and transferred to an analog signal by a D/A board (PCI-6052E, National Instruments, Austin, Texas, United States) and then played through a loudspeaker (K701, AKG, Vienna, Austria) at the top of recording box. The intensity of the sound stimulus was adjusted to be at 70 dB sound pressure level (SPL) and measured at the center of the recording box (Brüel and Kjær type 2,238 sound level meter, Naerum, Danish). In one session, 120 trials of click-train were presented at a random interval between 4 and 8s.

### IFN-α Treatment and Experiment Procedures

**FIGURE 1:**
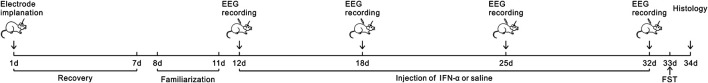
Experimental Design: the mice experienced surgery for electrode implantation and 7 days of recovery. The first EEG recording was conducted after 4 days of familiarization to the recording environments. Then IFN-α or saline was administered for 3 weeks. EEG recording was repeated at 7, 14, and 21 days after treatment. FST was conducted at the 21 days after EEG recording; then the brain was collected in the next day.

Recombinant human IFN-α was obtained from Miltenyi Biotec Inc. (Auburn, CA, United States). Stock solution of IFN-α was made up with distilled water into different aliquots containing 100,000 IU/ml. Prepared stock solutions were immediately stored at −20°C. Solutions for administration were prepared each day from these stock solutions, depending on the need for the day. As shown in the diagram of Figure 1, after completing one session of EEG recording under normal condition, the mice received a single subcutaneous injection of IFN-α (400 IU/g) or vehicle for 21 days.

Electrophysiological recording was conducted at 7, 14, and 21 days, respectively. At the 21st day after completing the EEG recording, animals were tested by the forced swimming test (FST) (Porsolt et al., 1977). The FST is a widely used measure of depressive-like behavior in rodents. Mice were placed into the glass cylinders (10 cm diameter) filled to a depth of 25 cm with water (25 ± 1°C) for 6 min before exposure. Behavior was video-recorded and later scored by an observer masked to treatment. The time of immobility (in seconds) was measured during the last 4 min of the 6 min period of exposure, leaving the first 2 min for habituation. An animal was judged to be immobile when it ceased struggling and remained floating motionless and making only movements allowing to keep the head just above the surface of water. They were then sacrificed in the next day and the brain tissue was processed by standard histological methods (see below).

### Electrophysiological Data Acquisition and Analysis

EEG signals were acquired through a flexible, low noise cable connected to the pin connector implanted on the skull of the mice. The microwire output was delivered to a multichannel preamplifier (PBX Preamplifier; Plexon, Dallas, Texas, United States) and then to a digital multichannel acquisition processor (MAP; Plexon). The waveforms of EEG were amplified and low-pass filtered with a 300 Hz cutoff frequency and then imported into MATLAB for analysis. First, EEG was visually checked to exclude the artifacts. The EEG fragments within an epoch of 500 m s before onset of a sound stimulus and 500 m s after stimulus offset were averaged for all trials without artifacts. The trial based spectra of EEG fragments were accessed by the mean trial power (MTP) analysis using a wavelet-based analysis algorithm, implemented in custom-written code using eeglab toolbox (https://sccn.ucsd.edu/eeglab/index.php). MTP was computed by averaging the EEG power in the spectral-temporal domain across the 120 trials from one session. The results of MTP were presented following a dB baseline correction implemented by eeglab.

### Immunofluorescence

Mice were anesthetized and transcardially perfused with 10 mM PBS, pH 7.5 at 4°C, followed by a fixative solution containing 4% PFA in PBS. Brains were postfixed in 4% PFA overnight at 4°C and cryoprotected for 72 h in 30% sucrose at 4°C before freezing in OCT on dry ice. A series of four coronal sections of the right hemisphere of hippocampus or AC was mounted for immunofluorescence analysis and stained with neuronal nuclear antigen (NeuN), ionized calcium-binding adapter molecule 1 (Iba-1), and glial fibrillary acidic protein (GFAP). In brief, brain sections were first blocked with 10% blocking serum in PBS and then incubated with the indicated primary antibodies (1:500 with anti-NeuN ab177487, 1:200 with anti-Iba1 ab178847, or 1:500 with anti-GFAP ab7260 from Abcam) overnight at 4°C. Slides were then incubated with secondary antibody for 2 h at room temperature. Rabbit highly cross-adsorbed AlexaFluor 594 secondary antibody (1:300, SA00006-8, Proteintech) was used to detect NeuN, Iba-1, or GFAP, respectively.

To minimize any potential confounding effects from immunohistochemistry, the sections were prepared, stained, and imaged at the same time as their relevant control. Furthermore, the cell number was counted in a predefined area of the brain. Nine sections among the serial coronal sections of the AC and hippocampus were selected from each brain, which were separated by 10 sections (50 μm). The areas of the AC and hippocampus were captured using an Olympus BX51 automatic microscope (Tokyo, Japan). The total numbers of cells stained with NeuN, Iba1, or GFAP in a 500 × 500 μm area were marked by an operator who was blinded to the identity of the sections, and an automated cell count was generated using ImageJ software (National Institutes of Health, Bethesda, MA, United States, http://rsb.info.nih.gov/ij/). Only morphologically intact and clearly identifiable cells were counted in the regions. The number of cells in each section was averaged to obtain a mean value for each animal (nine sections/mouse). The mean values obtained from ten animals in each group were used for the statistical analysis.

### Statistical Analysis

Statistical analysis was performed using SPSS for Windows (Chicago: SPSS, Inc.). All data are expressed as the group mean ± SEM. Values among multiple groups were analyzed using one-way repeated-measures ANOVA with Tukey’s post hoc test. The differences between two groups were calculated by a two-tailed unpaired *t*-test.

## Results

### Chronic IFN-α Treatment Reduced the ASSR of AC in Mice

**FIGURE 2:**
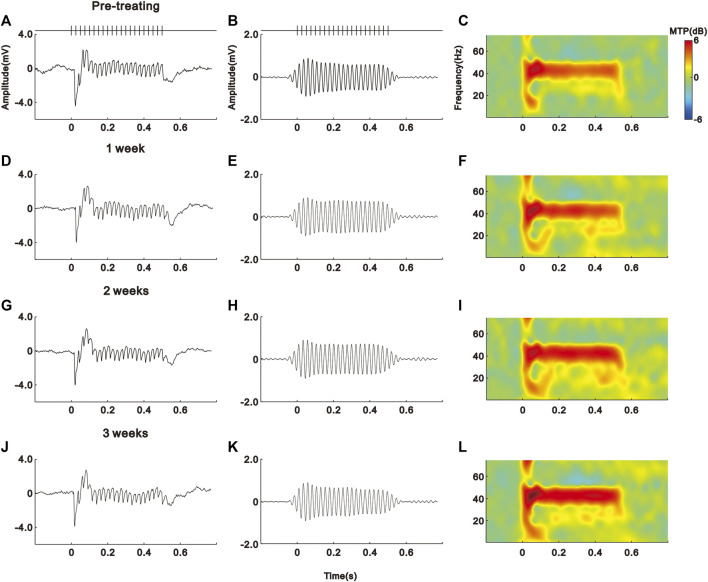
No effect of saline treatment on the ASSR of one example mouse. **(A)**–**(D)** Unfiltered ASSR averaged from 120 trials of EEG signals evoked by 40-Hz click stimuli lasting from 0 to 0.5 s. Bars at the top represent the sound wave of click-train stimulus. **(E)**–**(H)** The same EEG responses filtered with a bandpass filter. **(I)**–**(L)** Spectral-temporal spectrum of the filtered EEG responses.

**FIGURE 3:**
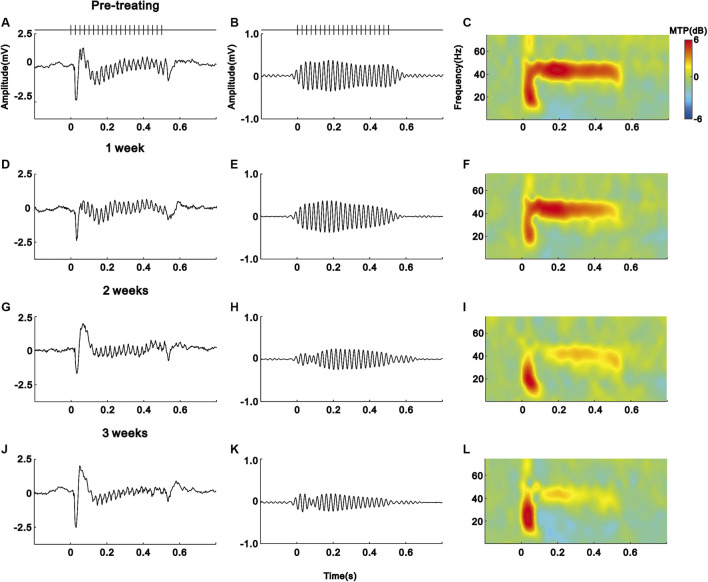
IFN-α treatment reduces the ASSR of one example mouse. Same format as Figure 2.

As shown in the schematic diagram of Figure 1, we subcutaneously injected saline (vehicle) or IFN-α in mice (*n* = 10 for each group) for 21 days. The EEG signals of each mouse were recorded before and during IFN-α treatment. The representative results of one saline and IFN-α treated mouse are presented in Figures 2 and 3, respectively. Before the treatment, EEG showed a large deflection at the onset of stimulus, followed by a stable oscillation synchronizing to the 40 Hz click-train (Figures 2A, 3A). To compare with the frequency of stimuli, the EEG signals were filtered with a bandpass filter of 35–45 Hz. The filtered EEG showed a clear oscillation synchronized to the stimulus frequency (Figures 2B, 3B). The power spectrum analyses on EEG also showed a clear peak at 40 Hz, reflecting the strength of 40 Hz ASSR (Figures 2C, 3C). In the end of the first week after saline or IFN-α injection, the ASSR recorded from the same mice remained unchanged (Figures 2D–F, 3D–F). However, the ASSR was gradually reduced from the second to the third week after IFN-α injection (Figures 2G–L, 3G–L).

**FIGURE 4:**
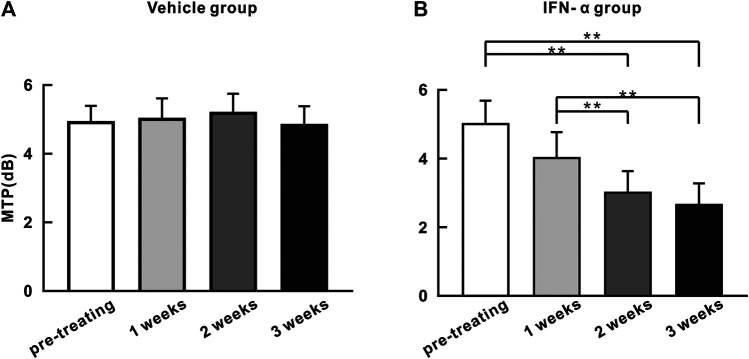
ASSR strength (MTP) does not significantly change with time in the saline injection group **(A)** but decreases with time in the IFN-α group **(B)**. Bars represent the mean of MTP (*n* = 10). Error bar represents SE. ** indicates *p* < 0.01, ANOVA, and Tukey’s post hoc test.

We quantified the strength of 40 Hz ASSR by calculating the average of MTP value in the spectral-temporal function between 35 and 45 Hz frequency range and during 0–0.55 s poststimulus time window. Figure 4 shows the mean MTP of the IFN-α and vehicle group (*n* = 10) at different time points. Injection of saline did not significantly change the ASSR (*p* = 0.445, one-way repeated-measures ANOVA, Figure 4A). In contrast, injection of IFN-α can significantly reduce the ASSR from the second week, comparing to pretreatment condition (*p* = 0.0001, one-way repeated-measures ANOVA and Tukey’s post hoc test, Figure 4B). Such a decrease continued to the third week (*p* = 0.0001, one-way repeated-measures ANOVA and Tukey’s post hoc test, Figure 4B).

### Correlation Between Reduction of ASSR and Depressive-Like Behaviors Induced by IFN-α

**FIGURE 5:**
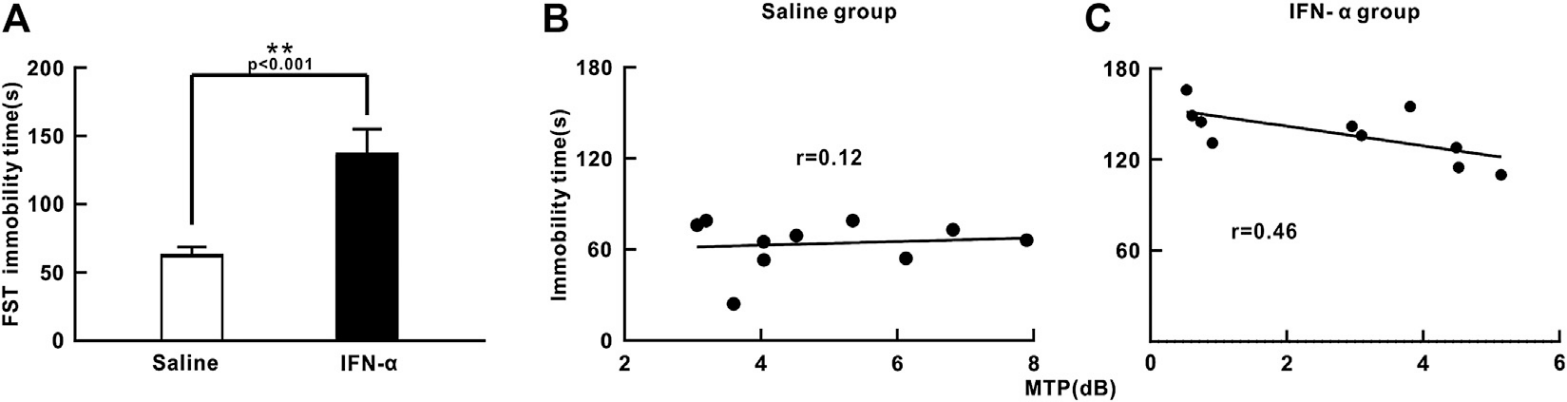
Effect of IFN-α treatment on immobility time during FST and correlation with ASSR. **(A)** IFN-α treatment increases immobility time in mice. *n* = 10 mice per group. Bars represent the mean ± SE. **p < 0.01, Student’s t-test. **(B)** No correlation between immobility time and MTP of ASSR in the saline injection group. **(C)** Significant correlation between immobility time and MTP of ASSR in the IFN-α injection group.

We next studied the effects of chronic IFN-α treatment on mouse behavior. Both the mice in the IFN-α and saline group had no significant difference of body weight between before and after treatment (IFN-α: 39.1 ± 2.9 g vs. 38.7 ± 2.8 g, *p* = 0.15, saline: 38.6 ± 1.9 g vs. 37.9 ± 1.7 g, *p* = 0.11, *n* = 10, *t*-test). Depression-like behaviors in these mice were examined using the FST (Porsolt et al., 1977). In the FST, depression levels are determined based on immobility times, which can be elongated by decreased escape-oriented behaviors. The immobility times were significantly increased by a 3-week treatment (*t* = 9.676, *p* = 0.0001, *df* = 18, *t*-test. Figure 5A), indicating that chronic IFN-a treatment induced depressive behavioral phenotypes, consistent with previous reports (Fahey et al., 2007; Felger et al., 2007). We further found no significant correlation between the immobility time and the power of 40 Hz ASSR in saline injection group (*r* = 0.12, *p* = 0.75, Pearson correlation, Figure 5B), but a negative correlation (*r* = 0.46, *p* = 0.03, Pearson correlation, Figure 5C) in the IFN-α injection group. This result suggests that ASSR can be used as an EEG marker of the depressive-like behavior in the IFN-α treated mouse.

### Chronic IFN-α Treatment Induces Glia Activation in AC and Hippocampus

**FIGURE 6:**
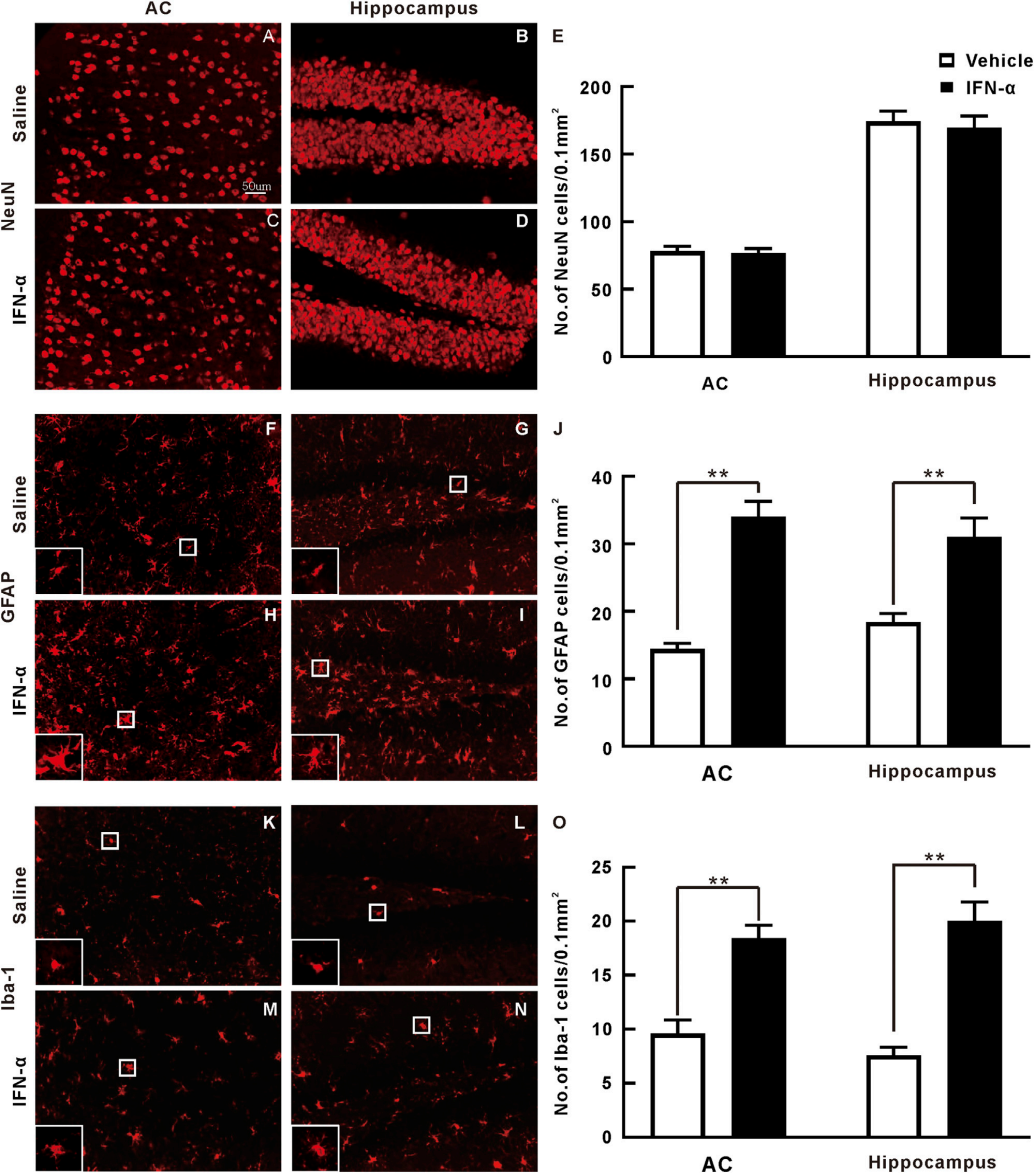
Immunohistochemistry analysis of AC and hippocampus sections after 3 weeks of saline or IFN-α injection. **(A)**–**(D)** Representative NeuN staining of the AC and hippocampus. **(E)** Bar graph of the density of NeuN stained cells. Results are expressed as mean ± SE. **(F)**–**(I)** Representative GFAP staining. White rectangle is an enlarged view of one representative cell to display the detailed morphology. **(J)** Bar graph of the density of GFAP stained cells. **(K)**–**(N)** Representative Iba-1 staining. **(O)** Bar graph of the density of Iba-1 stained cells. **p < 0.01, *t*-test.

We further found that the immunohistochemistry of NeuN in the AC and hippocampus was not obviously changed by IFN-α treatment as compared with vehicle (Figures 6A–D). Statistical analysis showed that there was no significant difference between the density of NeuN + neuron in the IFN-α and vehicle group (*t* = 0.896, *p* = 0.396 in AC; *t* = 0.298, *p* = 0.773, in hippocampus, df = 8, *t*-test, Figure 6E). However, IFN-α increased the density of astrocytes (*t* = 5.219, *p* = 0.0008 for AC; *t* = 4.938, *p* = 0.0011, for hippocampus, *df* = 8, *t*-test, Figure 6F, 6G, 6H, 6I, and 6J) and microglia (*t* = 7.209, *p* = 0.0001 for AC; *t* = 5.798, *p* = 0.0004, for hippocampus, df = 8, *t*-test, Figures 6K–O), accompanied by alterations of morphology. Thus, IFN-α treatment resulted in glial activation.

## Discussion

In the present study, we used a mouse model of chronic IFN-α treatment to demonstrate the effect of IFN-α on the neuroelectrical activity in CNS. The key findings are that chronic IFN-α treatment reduced the power of 40 Hz ASSR in the mouse AC, and the reduction of ASSR was accompanied with depressive-like behavior and glial activation in the AC and hippocampus. Our results suggest that EEG measurement of ASSR can be used as a biomarker to monitor the CNS side effects of IFN-α treatment and to search a novel intervention with potential therapeutic implications.

EEG is a minimally invasive method to assess brain activity. Previous studies on spontaneous EEG have reported some IFN-α induced alterations, such as enhanced slow-wave activity (Iivanainen et al., 1985) and increased synchronization (Birmanns et al., 1990). Recently, gamma band oscillations in EEG have become a subject of increasing research interest. However, effects of IFN-α on gamma band oscillations have not been examined; thus, we evaluated the brain activity with the usage of ASSR evoked by sounds with a gamma rhythm (40 Hz). ASSR, as an evoked gamma band oscillation, provides an approach for examining the synchronizing responses from large ensembles of neurons and binding neural activity across brain areas (Picton et al., 2003; Uhlhaas and Singer, 2010). ASSR paradigm is widely used in clinical studies of psychiatric disorders, including schizophrenia (Thune et al., 2016) and bipolar disorder (Uhlhaas and Singer, 2010; Oda et al., 2012; Isomura et al., 2016; Zhou et al., 2018). Depression is a major and serious side effect of IFN-α that limits its use as an antiviral and antitumor drug. In fact, IFN-α-induced depression-like behavior is one of the excellent models to explore the action of peripheral cytokine administration on CNS and to study the development of depression in a prospective way. Here, we for the first time reported that chronic IFN-α treatment in mice can induce a reduction of ASSR, which was moderately correlated with depressive-like behavior. Studies of the neurobiological basis of depression have focused on both principle excitatory glutamate neurons and inhibitory γ-amino butyric acid (GABA) interneurons. They demonstrate structural, functional, and neurochemical deficits in both major neuronal types that could lead to degradation of signal integrity in cortical and hippocampal regions (Duman et al., 2019). On the other hand, it has been reported that the ASSR is more sensitive to the modulation of glutamatergic transmission (Sullivan et al., 2015; Sivarao et al., 2016). Therefore, the abnormalities of ASSR observed in this study may be attributable to the dysfunction of glutamatergic transmission induced by IFN-α treatment. This possibility is worthy of further investigation.

In this study, we also found that 3 weeks of IFN-α treatment (400 IU/g/day) caused an increase of immobility time in FST. FST is a standard behavioral model to examine depression-like behavior, in which a prolongation of immobility represents behavioral despair (Petit-Demouliere et al., 2005). Our results are consistent with the previous studies showing that chronic application of IFN-α on rodent is an efficient way for establishment of behavioral despair accessed by the FST (Makino et al., 2000; Lv et al., 2018; Nicolussi et al., 2020). Furthermore, we found that the mice with a lower MTP of ASSR tended to show a longer immobility time, suggesting that ASSR can partly reflect the change of brain activity associated with the depressive-like behavior induced by IFN-α treatment. The increase of immobility time is also commonly reported in the experimental animals of depressive-like behavior induced by lipopolysaccharide (LPS) (Gu et al., 2018; Rodrigues et al., 2018; Arioz et al., 2019). Though EEG abnormalities have been examined in the LPS treated animals, all the previous studies focused on the changes of resting state EEG (Lin et al., 2010; Albrecht et al., 2018; Mamad et al., 2018). We, for the first time, investigated the association between the depressive-like behavior and EEG responses evoked by auditory stimuli. Our results suggest that EEG measurement of ASSR can be used as a biomarker to monitor the depressive-like behavior in animal models.

Our immunohistochemical results revealed that the density of NeuN + mature neurons in the AC was not affected by IFN-α treatment, but the density of astrocytes and microglia was significantly increased. Similar results were also found in the hippocampus, which is the most commonly studied brain region in depression research. Previous studies have suggested that dysfunction of neural plasticity in the hippocampus is involved in the neuropathology of depression (Zheng et al., 2015; Wachholz et al., 2016; Liu et al., 2017). Our results are consistent with the previous findings on hippocampus (Zheng et al., 2015; Wachholz et al., 2016). The limitation of our study is that we cannot clarify the underlying molecular and cellular mechanisms of the ASSR abnormality. For one thing, IFN-α may directly act on the neurons in the cortex and hippocampus, because the receptors of IFN have been found in the neurons (Owens et al., 2014; Zheng et al., 2014). On the other hand, the alterations of ASSR may be due to the changes of neuronal activity secondary to inflammatory process. IFN-α is known to induce the production of proinflammatory cytokines such as IL-1β, IL-6, and TNF-α (Wachholz et al., 2016). Elevated plasma concentrations of these proinflammatory cytokines have been reported in the IFN-α treated patients (Bonaccorso et al., 2001; Raison et al., 2010). Glial activation induced by the proinflammatory cytokines may disrupt the neural functions resulting in the ASSR abnormalities and depressive-like behaviors. Therefore, future research is necessary to determine the causality between the glial activation and ASSR alteration.

## Data Availability Statement

The raw data supporting the conclusions of this article will be made available by the authors, without undue reservation, to any qualified researcher.

## Ethics Statement

The animal study was reviewed and approved by the Animal Care and Use Committee of China Medical University.

## Author Contributions

YL was primarily responsible for experiment studies and statistical collection. ZL and XW assisted with statistical analysis; JC was responsible for literature search. LQ was primarily responsible for statistical analysis and manuscript editing. All authors contributed to and approved the final version of the manuscript.

## Funding

This work was supported by the grants from the National Nature Science Foundation of China under grant 31671080 to LQ, Department of Science and Technology of Liaoning Province (2017225024 and 2018225111 to PY), and Department of Science and Technology of Shenyang (19-109-4-15 to PY).

## Conflict of Interest

The authors declare that the research was conducted in the absence of any commercial or financial relationships that could be construed as a potential conflict of interest.

## References

[B1] Alavi M. Grebely J. Matthews G. V. Petoumenos K. Yeung B. Day C. (2012). Effect of pegylated interferon-α-2a treatment on mental health during recent hepatitis C virus infection. J. Gastroenterol. Hepatol. 27 (5), 957–965. 10.1111/j.1440-1746.2011.07035.x 22142332 PMC3331928

[B2] Albrecht M. A. Vaughn C. N. Erickson M. A. Clark S. M. Tonelli L. H. (2018). Time and frequency dependent changes in resting state EEG functional connectivity following lipopolysaccharide challenge in rats. PLoS One 13 (11), e0206985 10.1371/journal.pone.0206985 30418990 PMC6231634

[B3] Arioz B. I. Tastan B. Tarakcioglu E. Tufekci K. U. Olcum M. Ersoy N. (2019). Melatonin attenuates LPS-induced acute depressive-like behaviors and microglial NLRP3 inflammasome activation through the SIRT1/nrf2 pathway. Front. Immunol. 10, 1511 10.3389/fimmu.2019.01511 31327964 PMC6615259

[B4] Birmanns B. Saphier D. Abramsky O. (1990). Alpha-interferon modifies cortical EEG activity: dose-dependence and antagonism by naloxone. J. Neurol. Sci. 100 (1-2), 22–26. 10.1016/0022-510x(90)90007-a 1965206

[B5] Bonaccorso S. Puzella A. Marino V. Pasquini M. Biondi M. Artini M. (2001). Immunotherapy with interferon-alpha in patients affected by chronic hepatitis C induces an intercorrelated stimulation of the cytokine network and an increase in depressive and anxiety symptoms. Psychiatr. Res. 105 (1-2), 45–55. 10.1016/s0165-1781(01)00315-8 11740974

[B6] Borden E. C. Sen G. C. Uze G. Silverman R. H. Ransohoff R. M. Foster G. R. (2007). Interferons at age 50: past, current and future impact on biomedicine. Nat. Rev. Drug Discov. 6 (12), 975–990. 10.1038/nrd2422 18049472 PMC7097588

[B7] Calvet M. C. Gresser I. (1979). Interferon enhances the excitability of cultured neurones. Nature 278 (5704), 558–560. 10.1038/278558a0 431720

[B8] Dantzer R. O’Connor J. C. Freund G. G. Johnson R. W. Kelley K. W. (2008). From inflammation to sickness and depression: when the immune system subjugates the brain. Nat. Rev. Neurosci. 9 (1), 46–56. 10.1038/nrn2297 18073775 PMC2919277

[B9] De La Garza R. Asnis G. M. Pedrosa E. Stearns C. Migdal A. L. Reinus J. F. (2005). Recombinant human interferon-alpha does not alter reward behavior, or neuroimmune and neuroendocrine activation in rats. Prog. Neuro-Psychopharmacol. Biol. Psychiatry 29 (5), 781–792. 10.1016/j.pnpbp.2005.03.008 15927336

[B10] Dong C. Qin L. Liu Y. Zhang X. Sato Y. (2011). Neural responses in the primary auditory cortex of freely behaving cats while discriminating fast and slow click-trains. PloS One 6 (10), e25895 10.1371/journal.pone.0025895 21998717 PMC3187818

[B11] Dong C. Qin L. Zhao Z. Zhong R. Sato Y. (2013). Behavioral modulation of neural encoding of click-trains in the primary and nonprimary auditory cortex of cats. J. Neurosci. 33 (32), 13126–13137. 10.1523/JNEUROSCI.1724-13.2013 23926266 PMC6619728

[B12] Dowlati Y. Herrmann N. Swardfager W. Liu H. Sham L. Reim E. K. (2010). A meta-analysis of cytokines in major depression. Biol. Psychiatr. 67 (5), 446–457. 10.1016/j.biopsych.2009.09.033 20015486

[B13] Duman R. S. Sanacora G. Krystal J. H. (2019). Altered connectivity in depression: GABA and glutamate neurotransmitter deficits and reversal by novel treatments. Neuron. 102 (1), 75–90. 10.1016/j.neuron.2019.03.013 30946828 PMC6450409

[B14] Fahey B. Hickey B. Kelleher D. O’Dwyer A. M. O’Mara S. M. (2007). The widely-used anti-viral drug interferon-alpha induces depressive- and anxiogenic-like effects in healthy rats. Behav. Brain Res. 182 (1), 80–87. 10.1016/j.bbr.2007.05.005 17588681

[B15] Felger J. C. Alagbe O. Hu F. Mook D. Freeman A. A. Sanchez M. M. (2007). Effects of interferon-alpha on rhesus monkeys: a nonhuman primate model of cytokine-induced depression. Biol. Psychiatr. 62 (11), 1324–1333. 10.1016/j.biopsych.2007.05.026 PMC2149847 17678633

[B16] Gu M. Li Y. Tang H. Zhang C. Li W. Zhang Y. (2018). Endogenous omega (n)-3 fatty acids in fat-1 mice attenuated depression-like behavior, imbalance between microglial M1 and M2 phenotypes, and dysfunction of neurotrophins induced by lipopolysaccharide administration. Nutrients 10 (10), 1351 10.3390/nu10101351 PMC6213921 30248907

[B17] Iivanainen M. Laaksonen R. Niemi M. L. Färkkilä M. Bergström L. Mattson K. (1985). Memory and psychomotor impairment following high-dose interferon treatment in amyotrophic lateral sclerosis. Acta Neurol. Scand. 72 (5), 475–480. 10.1111/j.1600-0404.1985.tb00904.x 4082913

[B18] Isomura S. Onitsuka T. Tsuchimoto R. Nakamura I. Hirano S. Oda Y. (2016). Differentiation between major depressive disorder and bipolar disorder by auditory steady-state responses. J. Affect. Disord. 190, 800–806. 10.1016/j.jad.2015.11.034 26625092

[B19] Javitt D. C. Sweet R. A. (2015). Auditory dysfunction in schizophrenia: integrating clinical and basic features. Nat. Rev. Neurosci. 16 (9), 535–550. 10.1038/nrn4002 26289573 PMC4692466

[B20] Kim Y. K. Na K. S. Myint A. M. Leonard B. E. (2016). The role of pro-inflammatory cytokines in neuroinflammation, neurogenesis and the neuroendocrine system in major depression. Prog. Neuro-Psychopharmacol. Biol. Psychiatry 64, 277–284. 10.1016/j.pnpbp.2015.06.008 26111720

[B21] Kitagami T. Yamada K. Miura H. Hashimoto R. Nabeshima T. Ohta T. (2003). Mechanism of systemically injected interferon-alpha impeding monoamine biosynthesis in rats: role of nitric oxide as a signal crossing the blood-brain barrier. Brain Res. 978 (1-2), 104–114. 10.1016/s0006-8993(03)02776-8 12834904

[B22] Konsman J.-P. (2003). The mouse brain in stereotaxic coordinates. Psychoneuroendocrinology 28 (6), 827–828. 10.1016/s0306-4530(03)00088-x

[B23] Krishnan G. P. Hetrick W. P. Brenner C. A. Shekhar A. Steffen A. N. O’Donnell B. F. (2009). Steady state and induced auditory gamma deficits in schizophrenia. Neuroimage 47 (4), 1711–1719. 10.1016/j.neuroimage.2009.03.085 19371786 PMC2753273

[B24] Kumai T. Tateishi T. Tanaka M. Watanabe M. Shimizu H. Kobayashi S. (2000). Effect of interferon-alpha on tyrosine hydroxylase and catecholamine levels in the brain of rats. Life Sci. 67 (6), 663–669. 10.1016/s0024-3205(00)00660-3 12659172

[B25] Laguno M. Blanch J. Murillas J. Blanco J. L. León A. Lonca M. (2004). Depressive symptoms after initiation of interferon therapy in human immunodeficiency virus-infected patients with chronic hepatitis C. Antivir. Ther. 9 (6), 905–909. 10.1016/j.antiviral.2004.09.001 15651749

[B26] Leishman E. O’Donnell B. F. Millward J. B. Vohs J. L. Rass O. Krishnan G. P. (2015). Phencyclidine disrupts the auditory steady state response in rats. PloS One 10 (8), e0134979 10.1371/journal.pone.0134979 26258486 PMC4530939

[B27] Lin L. C. Chen Y. Y. Lee W. T. Chen H. L. Yang R. C. (2010). Heat shock pretreatment attenuates sepsis-associated encephalopathy in LPS-induced septic rats. Brain Dev. 32 (5), 371–377. 10.1016/j.braindev.2009.06.002 19574006

[B28] Liu W. Ge T. Leng Y. Pan Z. Fan J. Yang W. (2017). The role of neural plasticity in depression: from Hippocampus to prefrontal cortex. Neural Plast. 2017, 6871089 10.1155/2017/6871089 28246558 PMC5299163

[B29] Lv W. Booz G. W. Wang Y. Fan F. Roman R. J. (2018). Inflammation and renal fibrosis: recent developments on key signaling molecules as potential therapeutic targets. Eur. J. Pharmacol. 820, 65–76. 10.1016/j.ejphar.2017.12.016 29229532 PMC6733417

[B30] Maes M. (1999). Major depression and activation of the inflammatory response system. Adv. Exp. Med. Biol. 461, 25–46. 10.1007/978-0-585-37970-8_2 10442165

[B31] Makino M. Kitano Y. Komiyama C. Takasuna K. (2000). Human interferon-alpha increases immobility in the forced swimming test in rats. Psychopharmacology 148 (1), 106–110. 10.1007/s002130050031 10663424

[B32] Mamad O. Islam M. N. Cunningham C. Tsanov M. (2018). Differential response of hippocampal and prefrontal oscillations to systemic LPS application. Brain Res. 1681, 64–74. 10.1016/j.brainres.2017.12.036 29294350 PMC5792247

[B33] Mattson K. Niiranen A. Iivanainen M. Färkkilä M. Bergström L. Holsti L. R. (1983). Neurotoxicity of interferon. Canc. Treat Rep. 67 (10), 958–961. 6194882

[B34] Menzies R. Phelps C. Wiranowska M. Oliver J. Chen L. Horvath E. (1996). The effect of interferon-alpha on the pituitary-adrenal axis. J. Interferon Cytokine Res. 16 (8), 619–629. 10.1089/jir.1996.16.619 8877733

[B35] Miller A. H. Maletic V. Raison C. L. (2009). Inflammation and its discontents: the role of cytokines in the pathophysiology of major depression. Biol. Psychiatr. 65 (9), 732–741. 10.1016/j.biopsych.2008.11.029 PMC2680424 19150053

[B36] Musselman D. L. Lawson D. H. Gumnick J. F. Manatunga A. K. Penna S. Goodkin R. S. (2001). Paroxetine for the prevention of depression induced by high-dose interferon alfa. N. Engl. J. Med. 344 (13), 961–966. 10.1056/NEJM200103293441303 11274622

[B37] Na K. S. Jung H. Y. Kim Y. K. (2014). The role of pro-inflammatory cytokines in the neuroinflammation and neurogenesis of schizophrenia. Prog. Neuro-Psychopharmacol. Biol. Psychiatry 48, 277–286. 10.1016/j.pnpbp.2012.10.022 23123365

[B38] Nicolussi S. Drewe J. Butterweck V. Meyer Zu Schwabedissen H. E. (2020). Clinical relevance of St. John’s wort drug interactions revisited. Br. J. Pharmacol. 177 (6), 1212–1226. 10.1111/bph.14936 31742659 PMC7056460

[B39] Oda Y. Onitsuka T. Tsuchimoto R. Hirano S. Oribe N. Ueno T. (2012). Gamma band neural synchronization deficits for auditory steady state responses in bipolar disorder patients. PloS One 7 (7), e39955 10.1371/journal.pone.0039955 22792199 PMC3390322

[B40] Owens T. Khorooshi R. Wlodarczyk A. Asgari N. (2014). Interferons in the central nervous system: a few instruments play many tunes. Glia. 62 (3), 339–355. 10.1002/glia.22608 24588027

[B41] Petit-Demouliere B. Chenu F. Bourin M. (2005). Forced swimming test in mice: a review of antidepressant activity. Psychopharmacology 177 (3), 245–255. 10.1007/s00213-004-2048-7 15609067

[B42] Picton T. W. John M. S. Dimitrijevic A. Purcell D. (2003). Human auditory steady-state responses. Int. J. Audiol. 42 (4), 177–219. 10.3109/14992020309101316 12790346

[B43] Porsolt R. D. Bertin A. Jalfre M. (1977). Behavioral despair in mice: a primary screening test for antidepressants. Arch. Int. Pharmacodyn. Ther. 229 (2), 327–336. 596982

[B44] Quesada J. R. Talpaz M. Rios A. Kurzrock R. Gutterman J. U. (1986). Clinical toxicity of interferons in cancer patients: a review. J. Clin. Oncol. 4 (2), 234–243. 10.1200/JCO.1986.4.2.234 2418169

[B45] Raison C. L. Borisov A. S. Woolwine B. J. Massung B. Vogt G. Miller A. H. (2010). Interferon-alpha effects on diurnal hypothalamic-pituitary-adrenal axis activity: relationship with proinflammatory cytokines and behavior. Mol. Psychiatr. 15 (5), 535–547. 10.1038/mp.2008.58 PMC3403676 18521089

[B46] Raison C. L. Demetrashvili M. Capuron L. Miller A. H. (2005). Neuropsychiatric adverse effects of interferon-alpha: recognition and management. CNS Drugs. 19 (2), 105–123. 10.2165/00023210-200519020-00002 15697325 PMC1255968

[B47] Rodrigues F. T. S. de Souza M. R. M. Lima C. N. C. da Silva F. E. R. Costa D. Dos Santos C. C. (2018). Major depression model induced by repeated and intermittent lipopolysaccharide administration: long-lasting behavioral, neuroimmune and neuroprogressive alterations. J. Psychiatr. Res. 107, 57–67. 10.1016/j.jpsychires.2018.10.003 30326340

[B48] Rohatiner A. Z. Prior P. F. Burton A. C. Smith A. T. Balkwill F. R. Lister T. A. (1983). Central nervous system toxicity of interferon. Br. J. Canc. 47 (3), 419–422. 10.1038/bjc.1983.63 PMC2011317 6830692

[B49] Sammut S. Goodall G. Muscat R. (2001). Acute interferon-alpha administration modulates sucrose consumption in the rat. Psychoneuroendocrinology 26 (3), 261–272. 10.1016/s0306-4530(00)00051-2 11166489

[B50] Schiepers O. J. Wichers M. C. Maes M. (2005). Cytokines and major depression. Prog. Neuro-Psychopharmacol. Biol. Psychiatry 29 (2), 201–217. 10.1016/j.pnpbp.2004.11.003 15694227

[B51] Shahriari Y. Krusienski D. Dadi Y. S. Seo M. Shin H. S. Choi J. H. (2016). Impaired auditory evoked potentials and oscillations in frontal and auditory cortex of a schizophrenia mouse model. World J. Biol. Psychiatr. 17 (6), 439–448. 10.3109/15622975.2015.1112036 26796250

[B52] Sivarao D. V. Chen P. Senapati A. Yang Y. Fernandes A. Benitex Y. (2016). 40 Hz auditory steady-state response is a pharmacodynamic biomarker for cortical NMDA receptors. Neuropsychopharmacology 41 (9), 2232–2240. 10.1038/npp.2016.17 26837462 PMC4946051

[B53] Su K. P. Huang S. Y. Peng C. Y. Lai H. C. Huang C. L. Chen Y. C. (2010). Phospholipase A2 and cyclooxygenase 2 genes influence the risk of interferon-alpha-induced depression by regulating polyunsaturated fatty acids levels. Biol. Psychiatr. 67 (6), 550–557. 10.1016/j.biopsych.2009.11.005 PMC2982743 20034614

[B54] Sullivan E. M. Timi P. Hong L. E. O’Donnell P. (2015). Effects of NMDA and GABA-A receptor antagonism on auditory steady-state synchronization in awake behaving rats. Int. J. Neuropsychopharmacol 18 (7), pyu118 10.1093/ijnp/pyu118 25556198 PMC4540097

[B55] Suter C. C. Westmoreland B. F. Sharbrough F. W. Hermann R. C. (1984). Electroencephalographic abnormalities in interferon encephalopathy: a preliminary report. Mayo Clin. Proc. 59 (12), 847–850. 10.1016/s0025-6196(12)65620-1 6503366

[B56] Tarhini A. A. Gogas H. Kirkwood J. M. (2012). IFN-α in the treatment of melanoma. J. Immunol. 189 (8), 3789–3793. 10.4049/jimmunol.1290060 23042723 PMC4420629

[B57] Thuné H. Recasens M. Uhlhaas P. J. (2016). The 40 Hz auditory steady-state response in patients with schizophrenia: a meta-analysis. JAMA Psychiatry 73 (11), 1145–1153. 10.1001/jamapsychiatry.2016.2619 27732692

[B58] Uhlhaas P. J. Singer W. (2010). Abnormal neural oscillations and synchrony in schizophrenia. Nat. Rev. Neurosci. 11 (2), 100–113. 10.1038/nrn2774 20087360

[B59] Vial T. Descotes J. (1994). Clinical toxicity of the interferons. Drug Saf. 10 (2), 115–150. 10.2165/00002018-199410020-00003 7516663

[B60] Wachholz S. Esslinger M. Plümper J. Manitz M. P. Juckel G. Friebe A. (2016). Microglia activation is associated with IFN-α induced depressive-like behavior. Brain Behav. Immun. 55, 105–113. 10.1016/j.bbi.2015.09.016 26408795

[B61] Zheng L. S. Hitoshi S. Kaneko N. Takao K. Miyakawa T. Tanaka Y. (2014). Mechanisms for interferon-α-induced depression and neural stem cell dysfunction. Stem Cell Reports 3 (1), 73–84. 10.1016/j.stemcr.2014.05.015 25068123 PMC4110771

[B62] Zheng L. S. Kaneko N. Sawamoto K. (2015). Minocycline treatment ameliorates interferon-alpha- induced neurogenic defects and depression-like behaviors in mice. Front. Cell. Neurosci. 9, 5 10.3389/fncel.2015.00005 25674053 PMC4309184

[B63] Zhou T. H. Mueller N. E. Spencer K. M. Mallya S. G. Lewandowski K. E. Norris L. A. (2018). Auditory steady state response deficits are associated with symptom severity and poor functioning in patients with psychotic disorder. Schizophr. Res. 201, 278–286. 10.1016/j.schres.2018.05.027 29807805 PMC7003536

